# mRNA translation and protein synthesis: an analysis of different modelling methodologies and a new PBN based approach

**DOI:** 10.1186/1752-0509-8-25

**Published:** 2014-02-27

**Authors:** Yun-Bo Zhao, J Krishnan

**Affiliations:** 1 Department of Chemical Engineering, Centre for Process Systems Engineering, Institute for Systems and Synthetic Biology, Imperial College London, South Kensington, London SW7 2AZ, UK

**Keywords:** mRNA translation, Modelling methodology, Probabilistic Boolean network, Multiple-model methodology, Hybrid modelling

## Abstract

**Background:**

mRNA translation involves simultaneous movement of multiple ribosomes on the mRNA and is also subject to regulatory mechanisms at different stages. Translation can be described by various codon-based models, including ODE, TASEP, and Petri net models. Although such models have been extensively used, the overlap and differences between these models and the implications of the assumptions of each model has not been systematically elucidated. The selection of the most appropriate modelling framework, and the most appropriate way to develop coarse-grained/fine-grained models in different contexts is not clear.

**Results:**

We systematically analyze and compare how different modelling methodologies can be used to describe translation. We define various statistically equivalent codon-based simulation algorithms and analyze the importance of the update rule in determining the steady state, an aspect often neglected. Then a novel probabilistic Boolean network (PBN) model is proposed for modelling translation, which enjoys an exact numerical solution. This solution matches those of numerical simulation from other methods and acts as a complementary tool to analytical approximations and simulations. The advantages and limitations of various codon-based models are compared, and illustrated by examples with real biological complexities such as slow codons, premature termination and feedback regulation. Our studies reveal that while different models gives broadly similiar trends in many cases, important differences also arise and can be clearly seen, in the dependence of the translation rate on different parameters. Furthermore, the update rule affects the steady state solution.

**Conclusions:**

The codon-based models are based on different levels of abstraction. Our analysis suggests that a multiple model approach to understanding translation allows one to ascertain which aspects of the conclusions are robust with respect to the choice of modelling methodology, and when (and why) important differences may arise. This approach also allows for an optimal use of analysis tools, which is especially important when additional complexities or regulatory mechanisms are included. This approach can provide a robust platform for dissecting translation, and results in an improved predictive framework for applications in systems and synthetic biology.

## Background

mRNA translation is a ubiquitous process seen in almost all biological systems. In this process, the genetic codons are translated from mRNA to protein by ribosome translocation, after the genetic information contained in DNA is transcribed to the mRNA. The mRNA translation process involves three main players: the mRNA (genetic template), the ribosome (assembly machinery), and the aminoacyl transfer RNAs (aa-tRNAs), and is conceptually divided into three stages: initiation, elongation and termination. Specifically, the ribosome first attaches to the mRNA (initiation), reads the mRNA codon by codon (from the 5’ end of the mRNA to the 3’ end), recruits the appropriate aa-tRNA and knits the latest amino acid into the nascent peptide chain, releases the discharged tRNA (elongation), and finally releases the completed protein from the mRNA when the ribosome reaches the end of the mRNA (termination) [1]. mRNA translation follows broadly this same pattern in bacteria and eukaryotes with some differences in regulatory mechanisms.

Extensive studies on the mechanisms of mRNA translation have been reported and draw on multiple approaches and tools such as experimental cell biology, bioinformatics, theoretical and computational biology, and recently systems and synthetic biology [2-9]. However, even though the fundamental mechanisms underlying mRNA translation are relatively clear, a number of detailed regulatory mechanisms are only now being uncovered, the full understanding of which will require an interplay between experiments and modelling. Therefore, to elucidate the mechanism and functions of mRNA translation, a thorough, systems-level understanding is necessary, which consequently requires well-defined quantitative models. In addition, the understanding obtained from these quantitative models provides an important foundation for synthetic biology investigations [10-15].

The mathematical modelling of mRNA translation has a long history, and enjoys renewed interest in recent years with the development of systems and synthetic biology [16-28]. Models for mRNA translation are introduced with different formulations at various levels of abstraction, and can be divided into, roughly speaking, the ordinary differential equations (ODEs) based, and the Totally Asymmetric Simple Exclusion Process (TASEP) type models [29-31].

mRNA translation is the outcome of a number of transitions (which may be conceptualized as reactions), which can be typically modelled as a set of ODEs [32-39]. Such an ODE-based approach benefits from the extensive modelling and analysis tools available for ODEs. The ODE-based model usually treats each elongation step as one ODE (possibly multiple ODEs since each elongation step is the outcome of the interaction of multiple players including the mRNA, the aa-tRNA and several elongation factors [40]), and then the protein translation process is described in a comprehensive fashion [33,35]. However, the ODE-based model does not reflect some of the unique features of mRNA translation, that is, multiple ribosomes on an mRNA cannot simultaneously occupy one codon. As a result, in spite of their utility, the ODE-based models are not necessarily the default choice for modelling mRNA translation, although they are the dominant approaches in modelling other bioprocess such as gene transcription, signal transduction [41-43]. However, since mRNA translation primarily involves the ribosome movement along the mRNA, it can in many cases be studied without considering the detailed biochemical reactions/sub-processes. This simplified transportation problem can thus be modelled with TASEP, a model typically used in non-equilibrium physics [17,23,44-48], to quantitatively understand the particle transport in a one-dimensional lattice. Though simplified, the TASEP-based models have been used for obtaining such steady state information as the average occupancy of each codon on the mRNA, the mRNA translation rate, which are key in understanding mRNA translation. Finally, though not often seen, other methodologies exist for modelling mRNA translation, for example, a simplified deterministic Petri net based model which regards the initiation, elongation and termination events in mRNA translation as transitions in a timed Petri net [49], and a simplified version of TASEP named “ribosome flow model” where the codons on the mRNA are coarse-grained into larger segments [50,51].

As far as the whole process of mRNA translation is concerned, codon-based modelling, i.e., models that include the ribosome dynamics at each codon on the mRNA, is necessary and desirable. All these models, the ODE-based, the TASEP based, and the Petri net based, can be used in this way, but with different advantages and disadvantages. It is thus necessary to examine all these modelling methodologies, for the purpose of finding the appropriate modelling methodology to address specific questions regarding translation and translation regulation. In this work we examine and compare codon based stochastic models, ODE models and Petri net models. We try to rigorously define the codon-based models and the related simulation algorithms, clarify different update rules implicitly assumed in these models. We also propose a novel probabilistic Boolean network (PBN) based model and compare all these methodologies. Finally, we examine how these models can be used for situations which involve additional complexities and how multiple methodologies can help us better understand the mRNA translation process. Taken together, this analysis provides a systematic modelling platform, for use in understanding the translation process, in multiple contexts.

## Results and discussions

We present our analysis in the following sequence: 1) the definition of codon-based models and an analysis of the simulation algorithms; 2) the effect of the update rules in codon-based models; 3) a new PBN model for mRNA translation; 4) a comparison of the different modelling methodologies with added biological complexities; and 5) discussion. Further explanations on the numerical simulations and the related figures in this section can be referred to Additional file 1, the supplementary materials.

### The formulation, simulation and analysis of codon based models

#### Codon-based models are defined by both the rate law and the update rule

**Figure 1:**
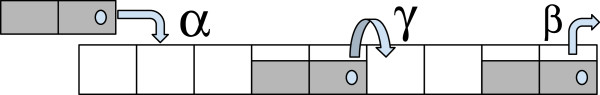
**The codon-based model for mRNA translation.** The codon-based lattice model for mRNA translation. The mRNA is represented by a one-dimensional lattice with each site being one codon on the mRNA. The ribosome attaches the leftmost end of the mRNA (initiation, the entry event) at some rate *α*, hops one codon towards its right (elongation, the hopping event) at some rate *γ* (possibly codon dependent) and exits from the rightmost point (termination, the exit event) at some rate *β*. Multiple ribosomes can be on the mRNA simultaneously.

The schematic diagram of the codon-based model for mRNA translation is illustrated in Figure 1. The mRNA is represented by a one-dimensional lattice with each site being one codon (triplet of nucleotides) on the mRNA, and the translation process involves the ribosome movement along the mRNA. This movement consists of three different types of events, corresponding to the three stages in mRNA translation, i.e., the entry of the ribosome from the leftmost region of the mRNA (initiation), the hops of the ribosome one codon a time to its right (elongation) and the exit of the ribosome from the rightmost region of the mRNA (termination). It is assumed that for a ribosome to attach the mRNA, the first *r* codons (which is the number of codons that a ribosome covers) must be empty, and for the ribosome to exit, its head must be at the last codon of the mRNA [29]. Various minor variations of this model are possible, and indeed it is also possible to mechanistically describe each movement step in more detail, but we will employ such a model in our analysis, as this has the main features relevant to the basic description of translation.

Three distinct features can be observed from the above codon-based model. First, each codon on the mRNA can be occupied by no more than one ribosome. Second, the ribosome can hop in only one direction. Third, multiple non-overlapping ribosomes can be on the mRNA simultaneously.

We now define the model in detail. The following notation is used in order to describe the model rigorously. The state of the *n* codons on the mRNA, or the “mRNA state”, is denoted by a vector *x*=[*x*_1_ … *x*_*n*_] where *x*_*i*_=1 means the *i*th codon is occupied by a ribosome and *x*_*i*_=0 the *i*th codon empty. An event is identified by the position of the head of the ribosome when the event occurs. The set of the possible events is then $E:=\{e_{i}:i\in I_{e}\}$ with the index set being $I_{e}:=\{0,r,r+1,\ldots ,n\}$, i.e., *e*_0_ the ribosome entry, *e*_*n*_ the ribosome exit, and *e*_*i*_,*i*=*r*,*r*+1,…,*n*−1 the ribosome hopping from codon *i* to *i*+1. Each event is associated with a rate, denoted by *α* for the entry, *β* for the exit and *γ*_*i*_,*i*=*r*,…,*n*−1 for the hops, respectively. We also use *γ*_0_ for *α*, *γ*_*n*_ for *β*, $\Gamma :=\{\gamma_{i}:i\in I_{e}\}$ being the set of the event rates for simplicity of notation.

The codon-based model can now be defined by specifying the event rates in *Γ* and the associated update rule. First, the event rates are interpreted as follows: within a time interval *dt*, the probability of event *e*_*i*_ to occur is *γ*_*i*_*dt* if the mRNA state at the time t allows such an event to occur; otherwise the probability is 0. For unknown mRNA state *x*, the actual event occurrence rate of event *e*_*i*_, denoted by $p_{e_{i}}(x)$, is dependent not only on the event rate *γ*_*i*_, but also on the event occurrence probability, denoted by *ψ*_*i*_(*x*), thus leading to the following rate law for the codon-based model,

(1) $$\begin{array}{l} p_{e_{i}}(x)=\psi_{i}(x)\gamma_{i} \end{array}$$

where the event occurrence probability is determined by the mRNA state *x*, as follows,

(2) $$\psi_{i}(x)=\left\{\begin{array}{l} P\{x_{j}=0,j=1,2,\ldots ,r\},\;i=0\\ P\{x_{n}=1\},\;\;\;\;\;\;i=n\\ P\{x_{i}=1,x_{i+1}=0\},\;i=r,r+1,\ldots n-1. \end{array}\right)$$

Second, the codon-based model is updated in discrete time steps and within one time step, more than one update event can be allowed since multiple ribosomes can be on the mRNA simultaneously. This fact thus implies that at any time step, an order of the update events, termed as the “update rule”, has to be specified. Although the update rule has to agree with the rate law in (1), more than one update rule can be possible and therefore which to choose has to be carefully determined.

Given the initial mRNA state (which is usually an empty mRNA, i.e., $x_{i}=0,i\in I_{c}$), the rate law (1) with the predetermined update rule defines completely the ribosome dynamics in Figure 1, thus yielding a complete definition of the codon-based (stochastic) model for mRNA translation.

#### The steady state of the codon-based model

The input parameters to the codon-based model are the initiation (entry), elongation (hopping) and termination (exit) rates (i.e. *Γ*) and the outputs of interest are often steady state information such as the codon density, which is defined as the average occupancy of the codons on the mRNA at the steady state, denoted by *ρ*_*i*_:=〈*x*_*i*_〉 for codon *i*, and the translation rate, which is defined as the average number of the translated proteins per unit time, denoted by *c*. For the basic codon-based model, the speed of the ribosome movement at each codon, or the average actual occurrence of each event, is the same at the steady state. Then the steady state can be characterized by

(3) $$p_{e_{i}}=\psi_{i}\gamma_{i}=c,i\in I_{e}$$

where $\psi_{i},i\in I_{e}$ are the average event occurrence probability at the steady state, i.e., *ψ*_*i*_=〈*ψ*_*i*_(*x*)〉.

This relationship is solely determined by the rate law but independent from the update rule. Indeed, *n*−*r*+3 variables $\left\{\psi_{i},c,i\in I_{e}\right\}$ are involved in the *n*−*r*+2 equations in (3). This marks the importance of the update rule in the sense that it determines the unique steady state solution given the event rates. Note that in the above model, we do not examine effects of ribosome limitation.

#### Codon-based models can be simulated by different algorithms

The codon-based models are analytically tractable for only very simple configurations. This makes the numerical simulations an important approach in the analysis of these models. The simulation algorithm is designed based on the rate law and the update rule which define the model. However, the rate law and the update rule do not, of course, determine a unique simulation algorithm. Therefore a careful investigation of simulation algorithms for the codon-based model is necessary. We present the following simulation algorithm which is applied throughout the paper, with more detailed discussions of alternative algorithms provided in the Methods Section. The presented algorithm uses a so-called random-sequential update rule which is the most widespread update rule employed in the literature. With this update rule, no particular order of the update events is assumed.

##### Algorithm 1 **Simulating TASEP with the random sequential update rule**

The parameters $p_{i}^{c}(x)$ and *dt* in Algorithm 1 are given in (14) and (15) in the Methods Section as follows: $p_{i}^{c}(x)=\frac{\gamma_{i}}{\underset{i\in I_{e}(x)}{\sum }\gamma_{i}}$ and $\text{dt}=\frac{1}{\underset{i\in I_{e}(x)}{\sum }\gamma_{i}}$ where $I_{e}(x)$ represents the set of all the allowed update events with mRNA state *x*. Then for the events that are allowed and not allowed to occur with mRNA state *x*, the average number of event occurrences during unit time are $p_{i}^{c}(x)/\text{dt}=\gamma_{i}$ and 0, respectively, which coincides with the rate law in (1). Hence, in terms of the long term steady state behaviour, this simulation algorithm can provide a well justified statistical approximation with arbitrary accuracy compared with the analytical solution to the model. Note that the update time steps in Algorithm 1 are time-varying with currently available update events, thus making its algorithm structure similar to the widespread Gillespie algorithms [52-54]. Other forms of alternative algorithms can be found in the Methods Sections. It is worth pointing out that simulations with other algorithmic variants resulted in the same steady states.

#### Different codon-based models lead to different steady state solutions

We compare the steady state solutions of three different codon-based models: ODE-based, TASEP-based and Petri net based. The TASEP-based model is simulated with the random sequential update rule as described in Algorithm 1, and the other two described below are analytically solved. 

• ODE-based model (Heinrich and Rapoport, 1980). Denote *ρ*_*m*_ the total concentration of mRNA in the considered volume, *ρ*_*r*_ the concentration of the free ribosomes, *h*_*i*_,*i*=*r*,…,*n* the average probability that on an mRNA codon *i* is occupied by the head of a ribosome, and *c*_*i*_ the corresponding flux for the ribosome movement from codon *i* to *i*+1 (in particular, *c*_0_ and *c*_*n*_ for the fluxes of ribosome entry and exit, respectively). The fluxes *c*_*i*_ can be determined as follows,

$$\begin{array}{lll} c_{0} & =\gamma_{0}\rho_{m}\rho_{r}W_{0}\; & \;\\ c_{i} & =\gamma_{i}\rho_{m}h_{i}W_{i},i=r,\ldots ,n\; & \; \end{array}$$

• where *W*_*n*_=1, *W*_0_ is the probability of the first *r* codons being empty, and *W*_*i*_,*i*=*r*,…,*n*−1 the conditional probabilities that codon *i*+1 is empty given that codon *i* is occupied by the head of a ribosome. Except *W*_0_ and *W*_*n*_, the conditional probabilities *W*_*i*_,*i*=*r*,…,*n*−1 cannot be determined directly by the given information. In the Heinrich model [32], they are calculated as follows with additional assumptions, the details of which can be referred to in the Methods Section,

(4) $$\begin{array}{c} W_{i}=\left\{\begin{array}{c} 1-\sum_{s=r}^{\min\{2r-1,n\}}h_{s},\;i=0\\ \frac{1-\sum_{s=1}^{r}h_{i+s}}{1-\sum_{s=1}^{r-1}h_{i+s}},\;r\leq i\leq n-r\\ 1,\;i=n-r+1,\ldots ,n \end{array}\right) \end{array}$$

**Figure 2:**
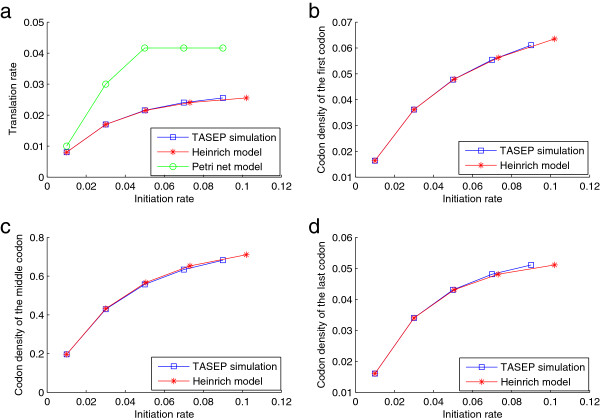
**Comparing ODE, TASEP and Petri net models.** The relationship between the initiation rate and **a)** the translation rate; **b)** the codon density of the first codon; **c)** the codon density of the middle codon; and **d)** the codon density of the last codon, as obtained from the various models: TASEP with the random-sequential update rule (Algorithm 1), the Heinrich model [32], and the deterministic Petri net model [49]. The parameters are set as *β*=*γ*_*i*_=0.5,*i*=*r*,…,*n*−1 (all parameters dimensionless). The waiting times in the Petri net model are determined by the mean of *Γ*, i.e., the inverse of the event rates, as discussed in [49]. The codon density is the average occupancy of the codons by the ribosome on the mRNA at the steady state.

• In Figure 2, in order to compare with the Petri net and TASEP models, we assume *ρ*_*r*_=1. Then the steady state of the Heinrich’s model is determined by the following equations

$$\begin{array}{l} \gamma_{0}W_{0}=\gamma_{i}h_{i}W_{i}=\gamma_{n}h_{n},i=r,\ldots ,n-1 \end{array}$$

• The relationship between the initiation, termination, elongation and translation rates is given by

$$\begin{array}{lll} h_{i} & =\frac{c}{\gamma_{i}},i=n-r+1,\ldots ,n\; & \;\\ h_{i} & =\frac{c(1-\sum_{s=1}^{r-1}h_{i+s})}{\gamma_{i}(1-\overset{r}{\underset{s=1}{\sum }}h_{i+s})},r\leq i\leq n-r\; & \;\\ \gamma_{0} & =\frac{c}{1-\sum_{s=r}^{\min\{2r-1,n\}}h_{s}}\; & \; \end{array}$$

• Further, noting that $h_{i}W_{i},i\in I_{e}$ is in fact the event occurrence probability *ψ*_*i*_, then the Heinrich’s model can be regarded as an approximation to the codon-based model by specifying $\psi_{i}=h_{i}W_{i},i\in I_{e}$. With this approximation, the *n*−*r*+2 independent variables $\psi_{i},i\in I_{e}$ are expressed by *n*−*r*+1 variables *h*_*i*_,*i*=*r*,…,*n*, and then the steady state Equations in (3) are solvable.

• Petri net model (Brackley et.al., 2012) [49]. A Petri net is a direct graph consisting of places (codons on the mRNA) and transitions (movement events of the ribosome). A transition is fired (an event occurs) if the corresponding places contain tokens (the mRNA state allows such an event to occur). In a timed Petri net, a waiting time is associated with the token, and the corresponding transition can be fired only if the associated waiting time has elapsed (the ribosome moves with the stochastic event rate).

• The Petri net model can be regarded as the deterministic analogue of the stochastic codon-based model (in a discrete event formulation), in the sense that the waiting times in the timed Petri net model are obtained from the deterministic mean of the stochastic event rates. The rate law implies that the average waiting time for the next event *e*_*i*_ to occur is exponentially distributed with parameter *γ*_*i*_. This thus leads to the fact that the average waiting time between two consecutive occurrence of event *e*_*i*_ is 1/*γ*_*i*_ provided that the mRNA state does not change during this time interval. Taking this into account, the Petri net model can then be defined by using 1/*γ*_*i*_ as the constant waiting time for transition *e*_*i*_ and mapping the conditions of event occurrence (its stochastic version is shown in (2)) into the tokens. This Petri net model has a very simple (discrete) deterministic ribosome dynamics which can be calculated exactly, for example, the translation rate is solely determined by the token(s) with the longest waiting time (the slowest event rate). The Petri net model has been studied analytically revealing this feature and the results match numerical simulation. For detailed descriptions of the Petri net model, and methods used to simulate as well as analyze this, the reader is referred to [49]. Note that in the original Petri net model, the ribosome covers only one codon. With multiple codon coverage, some modifications occur. As we have noted, the model requires the first r codons to be free for the ribosome to progress to start synthesizing proteins. Accordingly, the waiting times are modified to account for this. Thus we will employ an initiation waiting time which is determined by both the initiation rate and the sum of the first *r* elongation rates (whichever is slower). In this modified set up, we calculate the translation rate based on the slowest waiting time, in analogy with the results for the single codon coverage case.

The comparison of the steady states with these different models are illustrated in Figure 2. The following can be observed from the results. First, stochastic and deterministic interpretations of the event rates lead to distinct steady state solutions. A difference between the Petri net model and the other two models lies in the fact that the former is deterministic (with a discrete event formulation) while the latter two are based on stochastic event rates (the ODE is, of course, in a deterministic formulation). In Figure 2, both the translation rate and the codon density increase almost linearly with the initiation rate and saturate fairly quickly for the Petri net model (this is obtained as indicated earlier). These steady state profiles behave differently for the other two models.

Second, although the Heinrich model agrees with the TASEP-based simulations very well for slow initiation rates, there are numerical differences for larger values of initiation. Naturally, these conclusions may also vary, depending on the parameter regimes. It should be pointed out in Figure 2 that as the initiation rate increases beyond a threshold in the Petri-net model, the translation rate become insensitive to the initiation rate.

The different steady state solutions predicted by different models motivate the need for a comparative assessment of their relative merits and strengths.

### How is the codon-based model updated: the hidden assumption

In (3), the fact that as probabilities, $0<\psi_{i}<1,\forall i\in I_{e}$ implies that the translation rate is bounded between 0 and the minimum event rates (either initiation, elongation or termination). Hence, given the event rates, which unique steady state solution is finally determined is made by the update rule. This indicates that conclusions regarding the codon-based model should be made with explicit consideration of the update rule.

#### The parallel update rule for modelling mRNA translation

The update rules can be divided into two categories, non-ordered and ordered. The former, usually termed as “random sequential” and discussed earlier, does not assume any particular order of the update events. At any time step, the next event to be updated is chosen randomly among all the events with equal probability and updated probabilistically with its rate if the current state allows it to. Other rules that are not fully randomly updated contain, for example, sublattice-parallel, and ordered-sequential [55].

A particularly important ordered update rule is the parallel one. With this rule, at a specific time step, the update events occur to all that are possible to be updated. At first sight this rule seems to assume no particular order. However, in our codon-based models with only unidirectional transport, this update rule is in fact equivalent to the so-called particle-ordered-sequential update rule (with ordering starting from the left). With the latter, within a single time step, all the events that are allowed to be updated are updated probabilistically with their rates from the left to the right. The equivalence is immediately clear by noticing the fact that with the particle-ordered-sequential update rule, an event being updated on the left does not affect the update event to its right and therefore all the events that are allowed to be updated are actually updated probabilistically with their rates, as required in the parallel update rule.

Most codon-based models for mRNA translation use the random sequential update rule. This makes sense for two reasons. First, this update rule yields a much simpler master equation since the interactions in this update rule are for the nearby-neighbours only, and other update rules will usually lead to more non-local interactions. Second, simulating the random sequential update rule is also natural and straightforward. However, in reality, the ribosomes on the mRNA should act independently. A ribosome can move whenever its right codon is empty and this movement should not be affected by other ribosomes far away from it. This means that at any time step, all the possible movements of the ribosomes (or subsets thereof) could be allowed, without interactions between each other. This type of update is exactly what the parallel update rule does. In this sense the parallel update or variation thereof may be appropriate for modelling mRNA translation, as pointed out in [49] as well.

The simulation algorithm for the codon-based model with the parallel update rule is described in Algorithm 2, which benefits from its equivalence to the particle-ordered-sequential update rule. For mRNA state *x*, the probabilities of event $e_{i},i\in I_{e}(x)$ being updated is given as $p_{i}^{p}(x)=\frac{\gamma_{i}}{\underset{i\in I_{e}(x)}{\max}\gamma_{i}}$. Then the rate law implies that $\gamma_{i}dt^{p}=p_{i}^{p}(x)$, which further leads to the following time step

(5) $$dt^{p}=\frac{1}{\underset{i\in I_{e}(x)}{\max}\gamma_{i}}$$

The above definitions of $p_{i}^{p}(x)$ and *dt*^*p*^ ensure that given mRNA state *x*, the events that are allowed and not allowed to be updated are actually updated with rates $p_{i}^{p}(x)/dt^{p}=\gamma_{i}$ and 0, respectively. Therefore, although Algorithm 2 and Algorithm 1 are clearly different, both update rules still agree with the rate law defined for the codon-based model in (1).

##### Algorithm 2 **Simulating TASEP with the parallel update rule**

#### Different update rules give different steady state solutions

**Figure 3:**
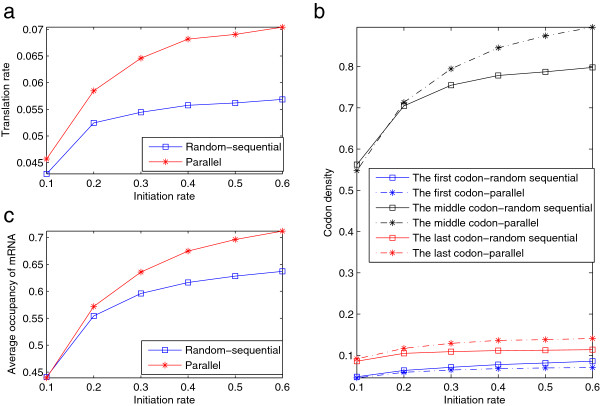
**Comparing the steady state solutions with different update rules.** The dependence on the initiation rate of the **a)** translation rate; **b)** codon density; and **c)** average occupancy of the mRNA, all at the steady state, for the random-sequential and parallel update rules, respectively. The parameters are set as *β*=0.5,*γ*=1. The codon density is the average occupancy of the codons by the ribosome on the mRNA at the steady state.

A comparison of the steady state solutions caused by the random-sequential and parallel update rules respectively, is provided in Figure 3. This comparison shows the relationship between the variations of the initiation rates and the steady state profiles. The differences between the two update rules can be observed as follows.

First, the parallel update rule generally leads to faster translation rates in all cases (Figure 3a). This observation will be further demonstrated by examples with added biological complexities in subsequent sections. From the random-sequential update rule in Algorithm 1 it is seen that one update event occurs within each time step $1/\underset{i\in I_{e}(x_{r})}{\sum }\gamma_{i}$ (where *x*_*r*_ is the mRNA state caused by the random-sequential update rule; *x*_*p*_ is used for the parallel update rule in what follows), making the translation rate being determined by $\langle \underset{i\in I_{e}(x_{r})}{\sum }\gamma_{i}\rangle$. On the other hand, the parallel update rule in Algorithm 2 means that within a time step $1/\underset{i\in I_{e}(x_{p})}{\max}\gamma_{i}$, the average number of the actually occurred update events is $\underset{i\in I_{e}(x_{p})}{\sum }\gamma_{i}/\underset{i\in I_{e}(x_{d})}{\max}\gamma_{i}$, thus making the translation rate being determined by $\langle \underset{i\in I_{e}(x_{p})}{\sum }\gamma_{i}\rangle$. Therefore, the fact that the translation rate with the parallel update rule is faster than the random-sequential one suggests that although all the possible mRNA states can be seen for both update rules, with the former, the mRNA state is such that those allowing more update events to occur are more often seen. In addition, it is also interesting to notice that the faster translation rate with the parallel update rule can also be an important contributory factor leading to much faster translation rate and higher codon density for the parallel updated Petri net model than the other two random-sequentially updated models in Figure 2.

Second, most codon densities exhibit the same pattern as the average number of ribosomes on mRNA (Figure 3b, 3c), since the latter is closely related to (principally determined by) the codon density, but this is not quite the case for either the leftmost nor the rightmost codons (Figure 3b). In fact, the higher translation rate of the parallel update rule makes a higher probability of the first codon being empty, meaning that the codon density of the first codon with the parallel update rule is usually smaller than that with the random-sequential one. On the other hand, a higher translation rate then naturally leads to a higher codon density of the last codon with the parallel update rule than that with the random-sequential one.

### Towards an exact steady state solution: probabilistic Boolean network based model

On the one hand, the analytical solution to the codon-based model for mRNA translation, which is often TASEP based, is available for only relatively simple configurations and analytical solutions with mean field approximations (or variations thereof) may be obtained in special cases; on the other, with only numerical simulations general conclusions and clear trends are not readily drawn. Therefore, a different approach for understanding the codon-based model could be interesting, especially when such an approach can partially bridge the gap between the analytical approaches to TASEP and numerical simulations. In this context, we present the PBN based model, discussed in detail as follows. Note that the following discussions are for the random-sequential update rule only.

#### mRNA translation can be modelled with PBN

A Boolean network is the dynamic interaction of multiple Boolean nodes where each node exhibits one of the only two states, 0 and 1. The evolution of the network state is governed by certain logical rules, or formally Boolean functions.

mRNA translation can be modelled within the Boolean network framework. Indeed, the mRNA state, *x*, is clearly Boolean, since each of its components (the codon state) can be only in state 0 or 1 and thus the codons can be regarded as Boolean nodes. Then the ribosome movement events that cause the dynamic evolution of the mRNA state corresponding to the Boolean functions. What makes mRNA translation different from a standard Boolean network is that the ribosome movement is probabilistic, governed by particular rate laws. This corresponds to a Boolean network where the governing Boolean function is chosen probabilistically from a set of candidates, and is formally referred to as a probabilistic Boolean network.

To formally describe the PBN model for mRNA translation, we introduce the following matrix expression of a Boolean function, in which a Boolean function is uniquely expressed as a linear system, as follows. How the matrix expression is derived and how various ribosome movement events are described in this form are described in the Methods Section.

$$x(t+1)=M_{x}x(t)$$

In the above expression, the mRNA state at time *t*+1, *x*(*t*+1), is dependent on both its state at time *t*, *x*(*t*), and the occurred ribosome movement event, described by the transition matrix *M*_*x*_. Notice that for *r*>1, the mRNA state space does not contain all the 2^*n*^ Boolean states. For example, for *n*=3, *r*=2, only 3 out of the 8 Boolean states are possible, i.e., [1 1 0], [0 1 1] and [0 0 0]. Therefore, the above dynamics is only applicable to the set of all the possibly allowed mRNA states for a specific pair of *n* and *r*.

The selection of the next event is probabilistic, i.e., for *M*_*x*_ being *M*_*i*_ it is associated with a probability *p*_*i*_, where *M*_*i*_ is to denote the transition matrix corresponding to event *e*_*i*_.

From the above, it is possible to define

$$M_{E}:=\underset{i\in I_{e}}{\sum }p_{i}M_{i}$$

where *M*_*E*_ denotes an averaged transition matrix, and its role will be seen below.

#### The steady state profiles can be numerically but exactly calculated with the PBN model

From the PBN theory the stationary distribution of the PBN model is fully determined by the transition matrix *M*_*E*_. Noticing that any two possible mRNA states are connected and any possible mRNA state can stay unchanged with a positive probability, we conclude that the underlying Markov chain governed by the transition matrix *M*_*E*_ of the PBN model is both irreducible and aperiodic. Therefore, the PBN model always leads to a stationary distribution which is the same as its asymptotic distribution. Denote this stationary distribution by *π*:=[*π*_1_*π*_2_ … *π*_*m*_]^*T*^ where *m* is the number of the possible mRNA states with specific *n* and *r*. Then *π* can be solved by either of the following two ways,

(6) $$M_{E}\pi =\pi ,\;\text{or}\;\left[\pi \;\pi \;\ldots \;\pi \right]=\underset{i\to \infty }{\lim}M_{E}^{i}$$

The stationary distribution *π* means that at the steady state, the probability of the mRNA state being the *i*th possible state, denoted by *χ*_*i*_, is given by *π*_*i*_. We call this probability, *π*_*i*_, the “state density” of mRNA state *χ*_*i*_.

The codon density and translation rate at the steady state can be calculated from the state density *π*, as follows,

(7) $$\begin{array}{l} \left[\rho_{1}\;\ldots \;\rho_{n}\right]=\overset{m}{\underset{i=1}{\sum }}\pi_{i}\chi_{i},\;c=\beta \rho_{n} \end{array}$$

Algorithm 3 describes the procedure for calculating the steady state profiles with the PBN model. Steps 2 and 3 can be automatically done by using the standard semi-tensor product toolbox for MATLAB [56] and therefore, given *n*, *r* and the event rates *Γ*, the steady state of the PBN model can be automatically calculated from Algorithm 3.

##### Algorithm 3 **Calculating the steady state profiles from the PBN model**

#### Analysis of the PBN model provides more information than TASEP models and enjoys exact steady state calculations

First consider a toy codon-based model with *n*=2 and *r*=1 to illustrate how the PBN model is constructed and analysed. Since *r*=1, all the four Boolean states are possible mRNA states. For the event rates of *α*=0.6, *β*=0.4, *γ*_1_=1, the transition matrix is obtained from Algorithm 3 as

$$M_{E}=\left(\begin{array}{cccc} 0.8000 & 0 & 0.3000 & 0\\ 0.2000 & 0.5000 & 0 & 0.3000\\ 0 & 0.5000 & 0.5000 & 0\\ 0 & 0 & 0.2000 & 0.7000 \end{array}\right)$$

From Algorithm 3, the state density can be calculated as *π*= [ 0.3600 0.2400 0.2400 0.1600], where the state densities are corresponding to the mRNA states *χ*_1_= [ 1 1], *χ*_2_= [ 1 0], *χ*_3_= [ 0 1] and *χ*_4_= [ 0 0], respectively. Then, the codon density and translation rate can be obtained by (7) as *ρ*= [ 0.6000 0.6000] and *c*=0.2400.

The codon density and the translation rate can be calculated by the analytical TASEP-based (or ODE) solution or TASEP-based simulations, while for the state density, one must employ either simulations or the PBN model. Although the codon density is a quantity often discussed in the literature, the state density contains more information and can be of great importance. For example, by using the information contained in the latter we are able to directly answer such questions as “the most and the least often seen mRNA states” (*χ*_1_= [ 1 1] and *χ*_4_= [ 0 0] respectively in the current example), which cannot be addressed by only the codon density.

The steady state profiles can also be obtained by numerical simulations. Running Algorithm 1 for 100000 time steps, the codon density and translation rate are obtained as *ρ*= [ 0.6008 0.6008] and *c*=0.2403. Compared to the exact solution provided by the PBN and analytical TASEP models, this level of error incurred by the numerical simulations might be acceptable. However, suppose for a specific mRNA translation process, we are interested in how the slight change of an event rate affects the steady state solution, i.e., the sensitivity of the event rate, then the accuracy of the steady state solution is vital since the calculation error could lead us to false conclusions. In this case, the PBN model with exact steady solution computation provides a suitable alternative to running TASEP simulations to estimate the asymptotic distribution (the TASEP model may not be able to give any analytical solution for complicated configurations).

### Examples with real biological complexities to elucidate the advantages and limitations of the models

In all the following examples (and examples presented above as well), the number of codons on the mRNA and the number of codons that the ribosome covers are set as *n*=50 and *r*=12, [17,32] for illustrative purposes. Simulations with 120 codons have also been performed, with similar conclusions. We employ this setting for illustrative purposes, so as to discuss the main points of interest. Since the mRNA state space is roughly exponentially increasing with *n* (the number of all the Boolean states for a network with *n* nodes is 2^*n*^ and the possible mRNA states are parts of them), a large number of *n*, e.g., several hundreds, could pose substantial computational challenges for solving the PBN model. On the other hand, the computational resources required by the TASEP-based simulations are also increasing rapidly with the increase of *n*. Increasing the computational efficiency of the steady state computation of the PBN model is ongoing work, and we discuss this issue in the conclusions.

#### Slow codons

The elongation rates are determined mainly by the concentrations of the corresponding tRNA and/or amino acids, and can thus be slowed due to rare corresponding tRNA and/or amino acids. These codons, termed as “slow” codons, become bottlenecks in the mRNA translation, and have severe effects on the steady state solutions [57-60].

**Figure 4:**
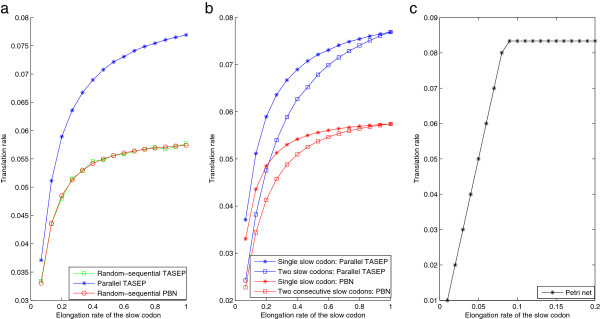
**The steady state translation rates with slow codons and different update rules.** The steady state translation rates for the case **a)** a single slow codon and different update rules; **b)** two consecutive slow codons; and **c)** slow codons and the Petri net model. The parameters are set as *α*=*β*=1. The elongation rates are also equal to 1 except the slow one(s). The cases of a single slow codon and two consecutive slow codons are considered, where for the first case the slow codon is on codon 25 and for the second they are on codons 25 and 26. In the second case, the two consecutive slow codons have the same elongation rate.

A comparison of the effects caused by the slow codons is illustrated in Figure 4, with multiple models including the Petri net model, the PBN model, TASEP-based simulations, and both the random-sequential and parallel update rules. Two observations are found. First, the steady state solution obtained by the TASEP-based simulations with the random-sequential update rule and the PBN model coincide with each other, proving the correctness of the PBN formulation. Second, in all cases the parallel update rule leads to faster translation rates than the random-sequential update rule, which further validates the conclusion made earlier in Figure 3.

It is reported in [57] that consecutive slow codons at the elongation stage (with the same elongation rate) give rise to slower translation rate than a single one, based on TASEP-based simulations with the random-sequential update rule. This is shown to be still valid in our simulations for the parallel update rule (Figure 4b). However, this effect cannot be predicted by the deterministic Petri net model (Figure 4c).Here the translation rate is determined by the slowest rate and the existence of either a single or multiple slowest rates do not matter. This point is briefly discussed in [49], and the authors offer an explanation to this effect by artificially introducing stochasticity to the event rates. The ODE model, e.g., the Heinrich model, on the other hand, does result in slower translation rate due to consecutive slow codons (results not shown).

In addition to the stationary translation rate, one may also be interested in the variations of the ribosome density across the mRNA, i.e., where and how long the queues of the ribosome are due to the existence of the slow codons. This information is of importance in optimizing protein expression. For this information, the PBN model may have particular value since, as mentioned earlier, the PBN model provides us with detailed and exact stationary ribosome distributions across the mRNA. The queues of the ribosome can then be predicted from this distribution, which however can be difficult for Petri net and TASEP models.

These results indicate that the appropriate model should be carefully chosen with respect to the problem to be investigated. To qualitatively understand the effects of a single slow codon, the Petri net model is acceptable. To include the effects of consecutive slow codons, TASEP-based models are sufficient, but for more quantitative understanding, the effects of different update rules have to be included. Finally, in order to know the specific mechanism that results in the slow codons, a comprehensive ODE model based on individual biochemical reactions is necessary.

#### Premature stop codons

mRNA translation is terminated via the recognition of the stop codon by the release factors. The nascent polypeptide is then released and folded to form functional proteins. In certain cases, through mutation or even by direct programming, in addition to the normal stop codon located at the end of the mRNA, a premature stop codon can be present in the mRNA. The transport of the ribosome thus bifurcates at the premature stop codon: it can either terminate here, thus resulting in truncated, potentially functionless, polypeptides, or readthrough the premature stop codon to produce the full-length protein (in some cases, the readthrough may be associated with a frameshift, but such details are somewhat tangential to the discussion here). Such a mechanism has been reported, for example, in the mRNAs encoding eRF1 and RF2 [6,39].

We will perform computational analysis of a scenario involving a premature stop codon where termination or readthrough may occur at the premature stop codon. Specifically, we assume for every ribosome encountering the premature stop codon, it can readthrough (thus proceed to the production of the full-length protein) with a fixed probability *μ* when the ribosome tries to move. The TASEP-based simulations can be readily modified to accommodate this change by adding the readthrough event to the set of events and adjusting the corresponding event rates. The PBN model can then be obtained from this new simulation algorithm, the details of which can be referred to the Methods Section.

**Figure 5:**
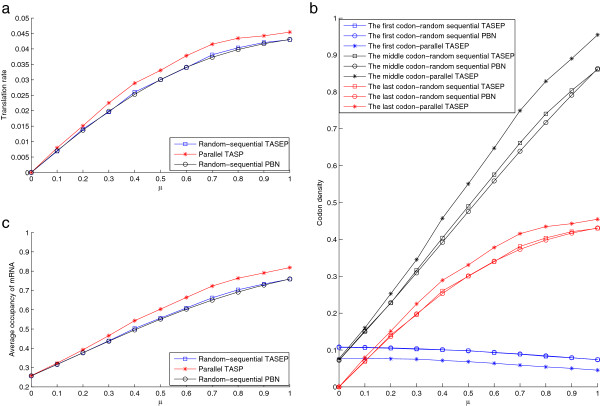
**The steady state solutions with the premature stop codon.** This figure shows the effect of propensity of readthrough (*μ*) at the premature stop codon and the corresponding **a)** translation rate; **b)** codon density; and **c)** average occupancy of the mRNA, all at the steady state. Both the parallel (TASEP simulations) and random-sequential update rules (PBN model and TASEP simulations) are considered. The parameters are set as *α*=1,*β*=0.1, $\gamma_{i}=1,i\in I_{e}$. The premature stop codon is at codon 25.

We show the steady state solutions with varying readthrough probability *μ* in Figure 5, for both update rules and both the PBN model and TASEP-based simulations. Again, as before, the parallel update still leads to faster translation rate in all cases. This is further evidence showing the importance of the update rules in the quantitative understanding of the mRNA translation process.

The translation rate is seen to be strictly increasing (almost linear) with the probability of readthrough for both update rules (Figure 5a), so are the average number of ribosomes on mRNA and most codon densities (Figure 5b, 5c). The decrease of the codon density of the first codon (Figure 5b) can be explained by the increase of the translation rate due to the increase of the probability of readthrough, as the faster translation rate makes the ribosome stay on the first codon shorter (similar explanations can be seen in Figure 3). These observations are not straightforward at first sight, especially that the probability of readthrough seems to dominates the overall translation rate in our simulations. This effect may be parameter-dependent, but at least in our case it shows the significant influence of the presence of the premature stop codon.

The effect of the premature stop codon cannot be easily directly modelled within the Petri net model framework, since at the premature stop codon, three ribosome movement events, i.e., staying where it is, stop codon readthrough and premature termination, exit rather than only the first two events for normal codons. Consequently, the existence of a third event makes the waiting times in the Petri net model undefined and so significant modification of the model structure is needed. We may simply treat the inclusion of the premature stop codon as an effect that slows the event rate at the premature stop codon (thus totally ignoring the premature termination event) by a factor *μ*. This results in a new waiting time at the premature stop codon as 1/*μγ*_*j*_. A similar linear increase of the translation rate with the increase of the probability of readthrough can be predicted if the translation rate is determined by the event rate at the premature stop codon (i.e. *γ*_*j*_ is the slowest event rate), but no similar predictions as in Figure 5 can be made otherwise. On the other hand, the Heinrich model may be possibly modified to accommodate the premature stop codon, while this modification will require the inclusion of an additional early termination event at the premature stop codon. We do not discuss this point further, except to note that the Heinrich model or related ODE models can be modified to incorporate premature stop codons and the readthrough rate (especially if rate limiting) can play a significant role in affecting the translation rate.

#### Negative autoregulation of initiation

mRNA translation is most likely to be regulated at the initiation stage in multiple situations, for a rapid control of gene expression at a low cost [7]. This regulation can be done by regulating the initiation factor activity (which affects almost all scanning-dependent initiation) and through sequence-specific RNA-binding proteins and microRNAs (which affect individual mRNAs), respectively. For example, it is suggested that the poly(A)-binding protein (PABP) is subject to a negative autoregulatory feedback loop where the overexpression of PABP leads to the autoregulatory repression of PABP itself [61]. Similar negative feedback mechanisms are also observed for initiation factors such as eIF1 and IF3 [6,62,63].

We perform a computational analysis of such a negative autoregulatory mechanism at the level of initiation. For this purpose, we describe this autoregulation mechanism with a simple model. To simplify the model, we assume that all the other factors that affect the initiation rate are kept unchanged, meaning that the variation of the initiation rate is solely determined by the concentration of the protein, denoted by *ρ*_*I*_, in a way that satisfies

(8) $$\alpha =\frac{1}{1+k_{I}\rho_{I}}\alpha_{I}$$

where *α*_*I*_ is interpreted as the maximum initiation rate (for *ρ*_*I*_→0), and *k*_*I*_>0 controls the autoregulation strength. We note that *α*→*α*_*I*_ for *k*_*I*_→0.

We now discuss two ways in which the feedback was incorporated.

The concentration of the protein is dependent on both its production rate, i.e., the translation rate *c*, and its degradation rate, denoted by *d*_*I*_ and assumed to be constant. In the first case, we will assume that *ρ*_*I*_ at steady state is given by the ratio of the translation rate to the degradation rate for instance, as the steady state of an evolution equation of the form *dρ*_*I*_/*dt*=*cρ*_*m*_−*d*_*I*_*ρ*_*I*_ where the concentration of the total mRNA *ρ*_*m*_ is assumed to be constant.

Note that in the above model, the feedback process from protein to initiation occurs in such a way that depends on the mean translation rate. The simultaneous solution of the equation (*ρ*_*I*_=*c*/*d*_*I*_) coupled with the equation for the translation process results in the steady state translation rate and protein concentration with such negative feedback present. If the steady state is stable, then its characteristics and the eventual state of the system can be obtained from this.

The second way of describing this system is to describe the production and degradation of the protein in an explicit stochastic description, coupled to the translation process. We have analyzed both models (which give essentially the same results) and will show results from the fully stochastic description. The full stochastic description is simulated for the random-sequential update rule using a modified version of Algorithm 1. In this modified algorithm, the change of the protein concentration is recorded at each step, which leads to the update of the initiation rate as in (8), and then the next update event is simulated based on a new set of event rates. Note that the evolution of *ρ*_*I*_ is simulated using its discrete-time version, where *dt* is corresponding to the varying update time steps as in Algorithm 1. We examine the model written above, to examine the role of feedback and related factors in affecting the steady state protein concentration.

**Figure 6:**
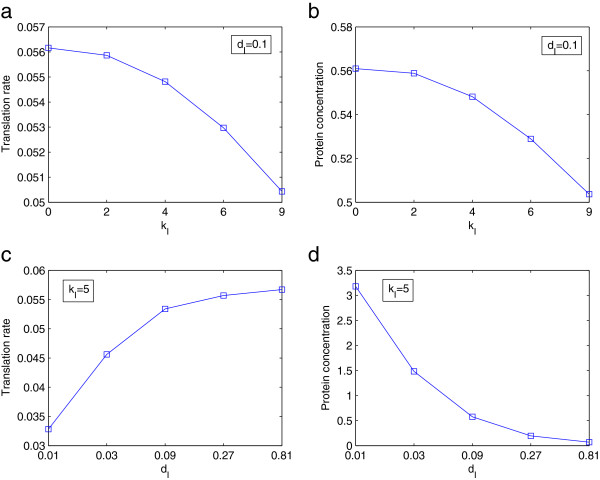
**The effect of negative feedback autoregulation.** The steady state for the case of negative feedback autoregulation detailed in (8), where **a)** and **b)** show the translation rate and protein concentration for fixed *d*_*I*_ and varying *k*_*I*_, while **c)** and **d)** correspond to fixed *k*_*I*_ and varying *d*_*I*_. The parameters are set as *β*=1, *γ*=1 and *ρ*_*m*_=1.

The steady state solutions governed by the autoregulation of the protein are shown in Figure 6. With the increase of the autoregulation strength *k*_*I*_ (note that *k*_*I*_=0 corresponds to the situation without autoregulation), the initiation rate decreases due to (8) (Figure 6a), which then leads to the decrease of the protein concentration *ρ*_*I*_ (Figure 6b). In fact, at the steady state the translation rate and the protein concentration are approximately proportional, which are verified from the similarity of the curves in Figure 6a and Figure 6b. On the other hand, the increase of the degradation rate *d*_*I*_ leads to the decrease of the protein concentration (Figure 6d), and then the increase of the initiation rate and consequently the translation rate (Figure 6c). Again, with respect to the protein concentration *ρ*_*I*_, the increase of the degradation rate *d*_*I*_ is a more dominant factor than the resulting increase of the initiation rate.

We also point out that the steady state translation rate and protein concentration can be solved from the first method by simulating the translation process for a given initiation rate, determining the translation rate, then determining the protein concentration and iterating. This amounts to “solving” for the steady state of the translation process with the negative feedback. In this context we also point out that a PBN formulation can be used to exactly obtain the translation rate for a given initiation rate (without simulation), and so can be used more effectively in the iterative process, than simulation. Thus numerically exact calculation of the steady states can also facilitate the solution of questions regarding how to tune feedback strengths to achieve particular steady state translation rates, and provides information about the state density in the presence of feedback.

Finally it is worth briefly examining the steady state of the Petri net model with feedback. We examine this as follows. We assume that protein concentration is determined as a linear function of the translation rate, exactly as above, and ask, what is the steady state protein concentration and translation rate from the Petri net model with feedback. Since the translation rate is determined by the slowest step, We see that if the initiation waiting time is not the dominant one, then the Petri net model reveals a translation rate which is insensitive to the feedback strength, for moderate levels of feedback. This is in contrast to TASEP, PBN and ODE formulations which do demonstrate sensitivity to the feedback strength (this is because the translation rate depends on the slowest rate in a rather simple way). Further, if the initiation is rate limiting, then the effect of (moderate) feedback in this model is stronger than in other models, because of the lack of “buffering” from other steps.

### Discussion

#### Modelling methodology comparisons

In this paper, we have analyzed different models of translation including their use to model regulatory phenomena, both in the simplest settings and with additional complexities incorporated. Models for mRNA translation can be divided into different categories based on their underlying assumptions. We briefly compare the different models in Table 1, and emphasize some relevant points, as follows. 

• The ODE models are deterministic, based on detailed biochemical reactions and do not enforce strict exclusion [34,35,64], TASEP-based models employ stochastic rate laws for ribosome movement in (1) and enforce strict exclusion [17,29,57], and the Petri net model assumes deterministic waiting times for ribosome movement, while enforcing exclusion as well [49].

• How the deterministic waiting times may be chosen a priori in more complex cases for the Petri net model is not clear. Further, the effect of parameter variation in Petri-net models is not as easy to analyze as ODE models, and this model appears to have limitations in systematically dealing with certain kinds of extra regulatory complexity in its current model structure.

• The numerically exact computation of the stationary state of the PBN models provides a useful complementary tool here.

• The number of the Boolean states is exponentially increasing with *n*, thus making the computational costs for the PBN model and TASEP-based simulations fast increasing with *n*. The use of iterative linear algebra Methods, will be useful in solving for steady states of PBN models when the size increases.

**Table 1:** Comparing codon-based models

	**Petri net**	**TASEP-based (PBN inc.)**	**ODE**
Assumption	Deterministic waiting times	Stochastic rate law	Biochemical reaction rates
Exclusion enforced	Yes	Yes	No
Update rule	Parallel	Random-sequential or parallel	N.A.
Solution methods	Analytical, simulations	Analytical, simulations, or numericallyexact (PBN)	Analytical, simulations, bifurcation analysis
Added complexity	Partly allowed	Allowed	Allowed

The PBN model provides exact steady state solutions compared to the statistical approximations of the TASEP-based simulations, and it is numerically solvable for models with real biological complexities which make the analytical approaches to TASEP difficult. Further, both in translation and elsewhere, the inclusion of the biological complexities, especially regulatory mechanisms, significantly modifies the TASEP structure. In systematically analyzing such modified TASEP structures, in some cases a complementary PBN may be useful, especially since there exist tools for analyzing both the PBN and its deterministic counterpart, using semi-tensor product formulations, going beyond simulations.

Our studies revealed that the models employed are in broad agreement in many cases, but that significant differences could be seen in the Petri net model both in the simplest model (Figure 2) and when additional variations such as slow codons are introduced (Figure 4). This should be mainly due to the different underlying assumptions between the Petri net model and TASEP-based models (and other ODE models): the former uses deterministic waiting times while the latter adopts stochastic ribosome movement rates.

The update rule is important in the quantitative understanding of the codon-based model for mRNA translation and its effects need to be accounted for. Most existing studies have focused on the random-sequential update rule [11,18,29,30]. It has been suggested however that a parallel-like update may be more appropriate for modelling mRNA translation [49]. The two update rules exhibit different behaviours for even the basic codon-based model as shown in Figure 3. These differences are more evident in the presence of real biological complexities. Although the examples presented in this study illustrates only quantitative differences, it is possible that update rules could lead to important qualitative changes when other form of biological complexities are included.

#### Multiple-model methodology and hybrid modelling for better understanding mRNA translation

The Petri net model, TASEP-based models and ODE models are models at different levels of abstraction with different assumptions made and relaxed, and give different insights. They also vary in the ease with which they can be thoroughly analyzed and dissected. Therefore the use of multiple modelling methodologies can provide a more complete understanding of the mRNA translation process, a robust platform from which to investigate specific biological effects. It also allows for an optimal use of available analysis tools. For instance, one can obtain some basic qualitative understanding from the Petri net or similarly based model, then uncover the structural properties from the TASEP-based models and finally probe specific regulatory problems with detailed ODE models. TASEP-based models are flexible and can accommodate real biological complexities (sometimes needing significant expansion/modification). In the TASEP-type models, the PBN model can be used to compute stationary distributions which may work effectively for moderate size problems; this can then be combined with numerical simulations for large systems, and finally analytical approaches to TASEP can be used, where possible, to give rise to more general conclusions. The exact computation of stationary distributions allows us to obtain important information about the stationary state and its sensitivity to parameters without doing repeated simulations. ODE models provide an easier framework for analysis, but do not naturally incorporate certain features such as strict exclusion. They can be analyzed much more easily than other models, especially when additional regulatory complexity is present and this becomes more pronounced as the model size increases. In addition, different tools from control engineering can be brought to bear here, something which is relevant in synthetic biology. The use of a combined model approach allows us to effectively combine tools of analysis on one hand with a handle on process complexity on the other (this is especially true of the TASEP/PBN/ODE models).

In addition, depending on the question under investigation, either fine grained (perhaps locally) or coarse grained models may be employed [50,51], and therefore it is important to be able to systematically fine-grain and coarse-grain models. Relatively coarse grained models have been shown to be useful, successfully making predictions in multiple contexts. The multiple model methodology may be useful here as well. For instance, certain coarse grained ribosome flow models, can be cast as and analyzed as probabilistic boolean models and their stationary distributions exactly numerically determined. This can be combined with models which incorporate more detailed resolution, which may be analyzed by simulation.

The above points highlight the tradeoff between the complexity of the model and the effectiveness in analyzing it. A basic aspect of interest in systems biology is what the role of intrinsic factors and parameters are and how they combine with extrinsic factors in regulating protein synthesis. One way to approach this is to employ suitable representations of the protein synthesis process and analyze this in silico. This includes the study of “synthetic genomes” [49] or the coupling with other factors. In synthetic biology it is desired to build robustly functioning circuits to meet particular objectives [10,65,66]. We see that in general ODE models allow for a detailed multiparametric sensitivity/bifurcation analysis. Detailed TASEP type models (possibly with significant extensions, incorporating finite pools of ribosomes, along with other factors) are analyzed primarily by simulations. A PBN type model (possibly coarse grained) can offer a simplified middle-ground model: it incorporates some of the essential features of translation, is stochastic, and can be used to perform multiparametric sensitivity analysis. This can be determined directly mathematically, once the stationary state is computed, and only needs matrix vector product computations. The result of such analysis can be used in conjunction with that of ODE models and detailed stochastic simulations.

In general the use of multiple methodologies in conjunction in specific problems, allows us to more clearly understand how different assumptions in the model, including those implicit in the modelling methodology, affect the conclusions and predictions. This in turn, would allow for a tighter set of conclusions which could be drawn and the effects of stochasticity, crowding and their interplay with regulatory complexity systematically elucidated with an effective use of available tools. This approach allows for predictions and extrapolations to be made with greater confidence.

It may be anticipated that in some situations a hybrid modelling approach can be useful: to model the mRNA translation process for a specific problem, those parts that are not directly related to the considered problem can be modelled with relatively simple descriptions and the parts which are the focus of interest are modelled in more detail. For example, to understand the autoregulation mechanism considered, the elongation and termination stages can be modelled with the TASEP assumptions (stochastic event rates) or simplifications thereof, while the initiation stage can be much more detailed (biochemical reactions).

This is equally relevant to understanding the natural coupling of translation with other processes. Finally it is important to be able to systematically and appropriately coarse grain models of translation. The use of multiple models in conjunction would be very helpful in making the transition from the individual process to the systems description.

## Conclusions

Translation is a basic genetic process which is widespread, and controlled in a multitude of ways in cells. Further the advent of synthetic biology suggests that there will be additional ways of this basic process being artificially regulated and manipulated [12,67]. The characteristic of translation is that it has a basic process (ribosome movement on mRNA) upon which is overlaid various additional regulatory and other complexities. Examples of this include regulatory mechanisms at initiation [5,20,36,68,69] and termination [70-73], nonsense mediated decay [39,74], the regulation of elongation steps by tRNA and the detailed mechanochemical steps involved in the ribosomal movement [22,40,75,76] and feedback [77]. Many of these aspects are being actively investigated experimentally. It is clear that modelling and computational frameworks need to be deployed in a systematic way to investigate many of these issues and to elucidate other issues such as the role of stochasticity in translation and its contribution to phenotypic noise.

Existing models of translation, already span a broad spectrum from the single ODE model to the detailed computational model of translation incorporating the effects of many factors [28]. The models we have examined and analyzed, exhibit an intermediate level of complexity, but are codon based. These models are built based on different assumptions of the mRNA translation process, thus making it important to clearly recognize the underlying assumptions and to select the right model(s) for specific problems. The different insights brought by different models also make the multiple-model methodology and hybrid modelling approaches desired choices for modelling and understanding the mRNA translation process. The multiple model methodology allows us to obtain a handle on process complexity on one hand and combine it with effective tools of analysis on the other. This is of relevance to both systems and synthetic biology.

### Systems biology

The understanding of translation and protein synthesis, and regulatory mechanisms therein, is an important theme in systems biology. Multiple data-driven models have been proposed for mRNA translation, where one is usually satisfied as long as the model matches the available experimental data (and possibly makes a few additional predictions successfully). However it is often the case that arbitrarily many models can be defined for the same data set and perform the same task, and further the applicability and limitations of the models are not systematically assessed. This means that the extent to which the models developed can be further employed is not clear. Nor is it clear, how different such models describing different facets of the system, actually fit together in effectively describing the full system.This makes it necessary for a careful investigation of the modeling methodologies and highlights the need for a systematic modelling approach involving multiple models and levels of description. In addition, the mRNA translation process is regulated at multiple levels which is related to other parts of the cellular system. Therefore, a detailed understanding of this process will then require such system level models as discussed in this work.

### Synthetic biology

A key aspect of synthetic biology is the precise control of gene expression and protein synthesis, and translation is an emerging area of focus. Synthetic biology is now engineering riboswitches, ribozymes, small RNAs [78,79], and other possible regulatory molecules to regulate protein synthesis, suggesting that sophisticated dynamic regulation of protein synthesis may be possible in the future. Thus far, the design has been done in a somewhat ad hoc and case-by-case manner, focusing on individual bio-blocks while lacking the system level understanding of the whole process. However the mRNA translation process is closely regulated at multiple levels and is also subject to noise, and further, synthetic circuits may be subject to extraneous interactions in the host cell(s). Therefore the system level understanding of the translation process itself and the different levels of regulation, is vital [14,15]. In addition, the models used for understanding, design and control purposes, should also be at an appropriate level of complexity, maintaining a balance between model complexity and the ability to analyse it (note that ODE models benefit from additional tools of control engineering), while making it possible to systematically account for and predict the effects of inherent regulatory effects and stochasticity. The modelling methodology comparison and analysis tools presented in this work provide powerful tools for this purpose, providing a useful foundation for synthetic biology.

Different models and different formalisms have been used in specific contexts to elucidate different aspects of translation in systems biology and design circuits in synthetic biology, and different levels of coarse and fine graining have been performed, all on a more-or-less ad hoc basis. Since in many cases the models describe different facets of the same system it is important to have a more unified and systematic framework which allows for a genuine systems understanding of the translation process as well as reliable simplifications thereof.

The approaches outlined above, possibly combined with tools such as Bayesian inference will allow for reliable and systematic frameworks, which both effectively distill the intrinsic complexity of translation, interaction with and control by extrinsic factors and can also be used with greater confidence for predictive purposes, as tools to complement experimental investigations, as well as for systems level descriptions. All these aspects provide substantial new challenges for modelling and computation of this basic genetic process which itself combines different scales and levels of complexity.

## Methods

In this section, we discuss different aspects of the models and simulation algorithms we employ. We discuss in turn (i) Some ODE models (ii) Simulation algorithms and variants for stochastic simulation of translation (iii)Formulation of Boolean rules to describe different events in translation.

### Heinrich’s model

We start by briefly discussing an ODE model developed by Heinrich and Rapoport.

Denote *ρ*_*m*_ the total concentration of the mRNA in the considered volume, *h*_*i*_,*i*=*r*,…,*n* the average probability that an mRNA codon *i* is occupied by the head of a ribosome, and *c*_*i*_ the flux for the ribosome movement from codon *i* to *i*+1 (in particular, *c*_0_ and *c*_*n*_ for the fluxes of ribosome entry and exit, respectively). The variation of the concentration of those mRNA whose *i*th codon is occupied by the head of a ribosome is determined by the following equations:

$$\begin{array}{lll} \rho_{m}\frac{dh_{i}}{\text{dt}} & =c_{i-1}-c_{i},i=r+1,\ldots ,n\; & \;\\ \rho_{m}\frac{dh_{r}}{\text{dt}} & =c_{0}-c_{r},\; & \; \end{array}$$

The fluxes *c*_*i*_ can be determined as follows. 

• *c*_*n*_. The ribosome can exit whenever its head is at the last codon of the mRNA. Therefore

$$\begin{array}{l} c_{n}=\gamma_{n}\rho_{m}h_{n} \end{array}$$

• *c*_0_. The ribosome can attach the mRNA whenever the first *r* codons on the mRNA are empty. Therefore,

$$c_{0}=\gamma_{0}\rho_{m}\rho_{r}W_{0}$$

where *ρ*_*r*_ is the concentration of the free ribosomes, and the probability of the first *r* codons being empty, denoted by *W*_0_, is given by

$$\begin{array}{l} W_{0}=1-\overset{\min\{2r-1,n\}}{\underset{s=r}{\sum }}h_{s} \end{array}$$

• *c*_*i*_,*i*=*r*,…,*n*−1. These fluxes can be generally written as

$$\begin{array}{l} c_{i}=\gamma_{i}\rho_{m}h_{i}W_{i},i=r,\ldots ,n-1 \end{array}$$

• where *W*_*i*_ is the conditional probability of codon *i*+1 being empty given codon *i* is occupied by the head of a ribosome.

• Without making further assumptions, *W*_*i*_ can not be determined. In the Heinrich’s model [32], an assumption is made that *W*_*i*_ equals the conditional probability of codon *i*+1 being empty given codon *i* is either empty or occupied by the head of a ribosome. The latter probability can then be shown to equal the conditional probability of codon *i*+1 being empty given codon *i*+1 is either empty or occupied by the tail of a ribosome [80,81], and can be calculated as

$$\begin{array}{l} W_{i}=\frac{1-\overset{r}{\underset{s=1}{\sum }}h_{i+s}}{1-\overset{r-1}{\underset{s=1}{\sum }}h_{i+s}},i=r,\ldots ,n-1 \end{array}$$

• Noting that *h*_*i*_=0,*i*>*n*, the above can further be written as

$$\begin{array}{lll} W_{i} & =\frac{1-\overset{r}{\underset{s=1}{\sum }}h_{i+s}}{1-\overset{r-1}{\underset{s=1}{\sum }}h_{i+s}},r\leq i\leq n-r\; & \;\\ W_{i} & =1,i=n-r+1,\ldots ,n-1\; & \; \end{array}$$

In Figure 2, in order to compare with the Petri net and TASEP models, we assume *ρ*_*r*_=1, i.e., a ribosome is always ready to start the initiation. Then the steady state solution is determined by the following equations

$$\gamma_{0}W_{0}=\gamma_{i}h_{i}W_{i}=\gamma_{n}h_{n}=c,i=r,\ldots ,n-1$$

The relation between the elongation termination and initiation rates and the translation rate is given by

$$\begin{array}{lll} h_{i} & =\frac{c}{\gamma_{i}},i=n-r+1,\ldots ,n\; & \;\\ h_{i} & =\frac{c(1-\overset{r-1}{\underset{s=1}{\sum }}h_{i+s})}{\gamma_{i}(1-\overset{r}{\underset{s=1}{\sum }}h_{i+s})},r\leq i\leq n-r\; & \;\\ \gamma_{0} & =\frac{c}{1-\overset{\min\{2r-1,n\}}{\underset{s=r}{\sum }}h_{s}}\; & \; \end{array}$$

Notice that $h_{i}W_{i},i\in I_{e}$ is in fact the event occurrence probability *ψ*_*i*_, then the Heinrich’s model can be regarded as an approximation to the codon-based model by specifying *ψ*_*i*_ as follows,

(9) $$\begin{array}{l} \psi_{i}=\left\{\begin{array}{ll} 1-\overset{\min\{2r-1,n\}}{\underset{s=r}{\sum }}h_{s}, & \;\;i=0\\ h_{i}\frac{1-\overset{r}{\underset{s=1}{\sum }}h_{i+s}}{1-\overset{r-1}{\underset{s=1}{\sum }}h_{i+s}}, & \;\;r\leq i\leq n-r\\ h_{i}, & \;\;i=n-r+1,\ldots ,n \end{array}\right) \end{array}$$

### The simulation algorithms

In this subsection, we briefly discuss simulation algorithms and their variants for simulating the basic translation process. All the following simulation algorithms are based on the rate law in (1) and the random-sequential update rule. With this update rule, no particular update order is predetermined: at each time step, the update event is chosen randomly with equal probabilities. For the sake of exposition, in what follows we assume that all the event rates are no more than one and can thus be interpreted as probabilities. This assumption does not lead to any loss of generality, as for any set of event rates $\Gamma =\{\gamma_{i},i\in I_{e}\}$, we can replace it by $\Gamma_{m}=\{\gamma_{i}/\underset{i\in I_{i}}{\max}\gamma_{i},i\in I_{e}\}$, and the steady state solution for the original event rates *Γ* can be obtained from the scaled *Γ*_*m*_ with the scale factor $\underset{i\in I_{i}}{\max}\gamma_{i}$.

We now discuss the algorithms and their variants. We begin with what may be regarded as a conventional algorithm [44]. 

• Conventional algorithm. The definition of the random-sequential update rule naturally leads to Algorithm 4, which clearly does not assume any particular update order. The time step *Δt* in Algorithm 4 is determined by making the algorithm fit with the rate law (1) and (2). From Algorithm 4, the number of the event occurrence of *e*_*i*_ within [*t*,*t*+*Δt*) should read $\frac{1}{n-r+2}\psi_{i}(x)\gamma_{i}$ where $\frac{1}{n-r+2}$ is the probability of the current update event being *i*. From (1) it thus holds that $\frac{1}{n-r+2}\psi_{i}(x)\gamma_{i}=\psi_{i}(x)\gamma_{i}\mathrm{\Delta t},i\in I_{e}$. Since this relationship holds for any [*t*,*t*+*Δt*) and *x*(*t*), therefore

(10) $$\mathrm{\Delta t}=\frac{1}{n-r+2}$$

• The determination of *Δt* in (10) ensures that within any time interval *Δt*, the actual event occurrence rate of Algorithms 4 is the same as what the rate law defines.

• This algorithm, picks out one possible update event chosen from a set of events with equal probability, checks if this event is possible, and if it is, probabilistically updates it in a manner commensurate with the event rate. This is described in more detail below.

• An alternative algorithm equivalent to Algorithm 4. Although Algorithm 4 is probably the most popular used algorithm in the literature, its structure does not readily allow modifications. We consider and discuss a variant Algorithm 5 for further discussions. The difference of these two algorithms lies in their algorithmic structures: Algorithm 4 first determines the update event with equal probability and then updates the event according to its rate, while Algorithm 5 combines these two steps by making the determination of the next update event directly dependent on their rates.

• As in Algorithm 4, Algorithm 5 does not predefine any particular update order and therefore it is still random sequentially updated. With Algorithm 5, the average number of occurrence of event *e*_*i*_ during [*t*,*t*+*Δt*) is $p_{i}^{d_{1}}\psi_{i}(x)$, which should equal *ψ*_*i*_(*x*)*γ*_*i*_*Δt* in Algorithm 4. Therefore, it yields that

(11) $$p_{i}^{d_{1}}=\frac{\gamma_{i}}{n-r+2}$$

• With the above discussion it is readily seen that Algorithms 4 and 5 are equivalent to each other.

• The efficient fixed-time-step algorithm. In Algorithm 5, the probability of an event index being chosen from $I_{e}$ is given by $\underset{i\in I_{e}}{\sum }p_{i}^{d_{1}}=\frac{\underset{i\in I_{e}}{\sum }\gamma_{i}}{n-r+2}<1$. This thus implies that Algorithm 5 (Algorithm 4 as well) skips a step without any action with probability $1-\underset{i\in I_{e}}{\sum }\gamma_{i}/(n-r+2)$. This is an obvious source of inefficiency.

• Algorithm 5 can be more efficient by replacing $\left\{p_{i}^{d_{1}}\right\}$ by

(12) $$p_{i}^{d_{2}}=\frac{\gamma_{i}}{\underset{i\in I_{e}}{\sum }\gamma_{i}},i\in I_{e}$$

• The more efficient algorithm is given in Algorithm 6. The new time step, *Δt*^′^, can be deduced from $p_{e_{i}}(x)=\frac{p_{i}^{d_{2}}\psi_{i}(x)}{\Delta t^{\prime }}=\frac{p_{i}^{d_{1}}\psi_{i}(x)}{\mathrm{\Delta t}},i\in I_{e},\forall x$, which gives

(13) $$\Delta t^{\prime }=\frac{1}{\underset{i\in I_{e}}{\sum }\gamma_{i}}$$

• The efficient varying-time-step algorithm. In Algorithms 5 and 6 the selected update event may not actually occur as the state may not allow it to. At the steady sate, the average probability that a time step is skipped can be determined for Algorithms 5 and 6 as $p_{d}:=1-\underset{i\in I_{e}}{\sum }p_{i}^{d}\psi_{i}\geq 0$ where $p_{i}^{d}$ can be $p_{i}^{d_{1}}$ or $p_{i}^{d_{2}}$, respectively. Notice that the sum of the probabilities of defining the next event index is always no more than one, i.e., $\underset{i\in I_{e}}{\sum }p_{i}^{d}\leq 1$. Therefore Algorithm 6 is already the most possible efficient algorithm with the same simulation procedure since $\underset{i\in I_{e}}{\sum }p_{i}^{d_{2}}=1$. Any improvement of the algorithm efficiency has to be made by modifying the algorithm structure. This is done by switching the fixed time steps in Algorithms 5 and 6 to time-varying ones in Algorithm 1, as follows.

• For a specific state *x*, denote the set of the indices of all the possible update events by $I_{e}(x)\subset I_{e}$ and the corresponding rates by $\Gamma (x):=\{\gamma_{i}:i\in I_{e}(x)\}\subset \Gamma$. $I_{e}(x)$ is entirely determined by the state *x* and is thus time-varying. Define the probability of the index of the next update event being $i\in I_{e}(x)$ by

(14) $$p_{i}^{c}(x)=\frac{\gamma_{i}}{\underset{i\in I_{e}(x)}{\sum }\gamma_{i}},i\in I_{e}(x)$$

• Note that the actual event occurrence rate of *e*_*i*_ is proportional to *γ*_*i*_ and the sum of all these probabilities equal one, i.e. $\underset{i\in I_{e}(x)}{\sum }p_{i}^{c}(x)=1$.

• Within the simulation time step, denoted by *dt*, for $i\notin I_{e}(x)$, the number of actual event occurrence is 0 and for $i\in I_{e}(x)$, it holds that $p_{i}^{c}(x)=\gamma_{i}\text{dt}$, which gives

(15) $$\begin{array}{l} \text{dt}=\frac{1}{\underset{i\in I_{e}(x)}{\sum }\gamma_{i}} \end{array}$$

• Therefore, the new algorithm given in Algorithm 1, still agrees with the rate law and the random sequential update rule. Note that the time steps in Algorithm 1 are time-varying with the current mRNA state.

• The statistical equivalence of the algorithms. Although the update time interval and update mechanisms are different, all the algorithms ensure that within their individual update time interval, the probability of event occurrence is exactly given as in (1), and the update order is not particularly determined (random-sequential). Therefore, in the long run all these algorithms are equivalent in the statistical sense, leading to the fact that all the statistical characteristics as the translation rate and codon density are the same for all the algorithms.

• On the other hand, at the steady state the average time interval between two consecutive events is $\langle \text{dt}\rangle =\langle \frac{1}{\underset{i\in I_{e}(x)}{\sum }\gamma_{i}}\rangle =\frac{1}{(n-r+2)c}$ (determined by Algorithm 1) but the simulation time intervals for different algorithms are $\mathrm{\Delta t}=\frac{1}{n-r+2}$ for Algorithms 4 and 5, $\Delta t^{\prime }=\frac{1}{\underset{i\in I_{e}}{\sum }\gamma_{i}}$ for Algorithm 6 and 〈*dt*〉 for Algorithm 1, respectively. Therefore, on the average $\langle \frac{n-r+2}{\underset{i\in I_{e}(x)}{\sum }\gamma_{i}}\rangle$ (or, $\frac{1}{c}$) time steps in Algorithm 4 and 5 would result in an actual update event, and $\langle \frac{\underset{i\in I_{e}}{\sum }\gamma_{i}}{\underset{i\in I_{e}(x)}{\sum }\gamma_{i}}\rangle$ (or, $\langle \frac{\underset{i\in I_{e}}{\sum }\gamma_{i}}{(n-r+2)c}\rangle$) time steps in Algorithm 6. Only for Algorithm 1 every time step will definitely result in an actual update event.

#### Algorithm 4 **The conventional algorithm with the random-sequential update rule**

#### Algorithm 5 **The equivalent alternative algorithm with the random-sequential update rule**

#### Algorithm 6 **The efficient fixed-time-step algorithm with the random-sequential update rule**

### The PBN model

The derivation of the PBN model is based on Algorithm 6 with the random-sequential update rule. As shown above, all the simulation algorithms with the same random-sequential update rule are statistically equivalent and therefore the choice of the underlying algorithm does not limit the PBN model in any sense. The PBN model with the parallel update rule is ongoing work and will not be discussed here. We first present some background and preliminary details on the PBN model and then discuss how the various events are represented in this setting.

In order to derive the PBN model, one must be able to first express the mRNA state as the state in a Boolean network and then the update events as Boolean functions governing the dynamics of the Boolean network. Conceptually this can be readily done as the mRNA codon state is indeed Boolean (a codon being covered or uncovered by a ribosome constitute its two Boolean states). However, historically no efficient tools for Boolean networks have been available, which may be a reason why this seemingly straightforward PBN model for mRNA translation has not been discussed before. In what follows, we show that based on a recently developed tool based on the “semi-tensor product”, Boolean networks can be represented as linear discrete systems, and consequently the mRNA translation process can be modelled as a PBN which can be rigorously formulated and computationally solvable.

#### The matrix representation of Boolean networks

A Boolean network is the dynamic interactions of multiple Boolean nodes where each node can be one of the only two possible states, thus making 2^*n*^ different states for the whole network. A Boolean network with *n* nodes can be generally represented as follows,

$$\begin{array}{l} \left\{\begin{array}{ll} x_{1}(t+1) & =f_{1}(x_{1}(t),\ldots ,x_{n}(t))\\ & \vdots \\ x_{n}(t+1) & =f_{n}(x_{1}(t),\ldots ,x_{n}(t)) \end{array}\right) \end{array}$$

where *x*_*i*_,*i*=1,…,*n* represent the nodes, and the dynamics of the nodes are governed by the Boolean functions *f*_*i*_,*i*=1,…,*n*.

Boolean networks are typically analysed using truth tables, i.e., tables listing all the possible one step state change caused by the Boolean functions. This approach of analysis is evidently not a powerful one but has however been the only one for a long time before the introduction of the matrix representation of Boolean networks based on the so-called semi-tensor product in recent years [82,83].

The semi-tensor product is an extended matrix product. For matrices *X* and *Y* with any dimensions *r*_1_×*c*_1_ and *r*_2_×*c*_2_, the semi-tensor product of *X* and *Y*, denoted by X⋉Y, is defined as follows,

(16) $$\begin{array}{l} X⋉Y:=(X⊗I_{\text{lcm}(c_{1},r_{2})/c_{1}})\times (Y⊗I_{\text{lcm}(c_{1},r_{2})/r_{2}}) \end{array}$$

where lcm(*c*_1_,*r*_2_) is the least common multiple of *c*_1_ and *r*_2_, ⊗ is the Kronecker product and × is the normal matrix product.

The most interesting aspect of the semi-tensor product in this context is, with it Boolean networks can be represented in a matrix form. Specifically, mapping logical “TRUE” and “FALSE” to $\delta_{2}^{1}$ and $\delta_{2}^{2}$, respectively, where $\delta_{n}^{k}$ generally represents the *k*th column of the identity matrix with dimension *n*, then for any Boolean function *f*(*x*_1_,*x*_2_,…,*x*_*n*_), a unique matrix *M* with 2^*n*^ columns and the columns being chosen from $\delta_{2}^{1}$ and $\delta_{2}^{2}$, called the structure matrix of *f*, exists and

(17) $$\begin{array}{l} f(x_{1},x_{2},\ldots ,x_{n})=M{⋉}_{i=1}^{n}x_{i} \end{array}$$

That is, any Boolean function can be uniquely identified by and equivalently treated with its structure matrix [83]. This provides a succinct and systematic way of representing the network, which can be systematically augmented. Additionally this representation provides new tools for analysis of attractors of deterministic analogues of such networks.

Therefore, let $x(t):={⋉}_{i=1}^{n}x_{i}(t)$ and the Boolean network can then be equivalently written as

$$\begin{array}{l} \left\{\begin{array}{ll} x_{1}(t+1) & =L_{1}x(t)\\ & \vdots \\ x_{n}(t+1) & =L_{n}x(t) \end{array}\right) \end{array}$$

where *L*_*i*_ is the structure matrix corresponding to function *f*_*i*_. This further leads to

(18) x(t+1)=Lx(t)

where *L*=*L*_1_∗*L*_2_∗…∗*L*_*n*_ and ∗ is the Khatri-Rao product. That is,

$${\text{Col}}_{i}(L)={⋉}_{j=1}^{n}{\text{Col}}_{i}(L_{j}),i=1,\ldots ,2^{n}$$

where Col_*i*_(*L*) is the *i*th column of *L*. That is, a Boolean network based on the logical rules is equivalent to a linear system in (18) and is completely described by the structure matrix *L*.

Finally, a Boolean network becomes a probabilistic one, i.e. a PBN, if the dynamics of the network is probabilistically determined, i.e., its structure matrix *L* can be chosen from a set of possible candidates () with certain probabilities (),

(19) $$P\{L=L^{\prime }\}=p^{\prime },L^{\prime }\in ℒ,p^{\prime }\in P$$

where $\underset{p^{\prime }\in P}{\sum }p^{\prime }=1$.

#### The Boolean network description of the update events

In this subsection, we discuss how the various events in the translation process may be described in Boolean terms. The three types of the update events in Algorithm 6 can be described as Boolean functions associated with the Boolean network consisting of the mRNA state *x*. As mentioned earlier, as long as we are able to formally describe the update events as Boolean functions (i.e., (20), (21) and (22)), their matrix representations can then be transformed automatically with the semi-tensor product toolbox [56]. Therefore in what follows we focus only on the Boolean expression of the update events but not their further calculations within the semi-tensor product framework.

The following logical operators are used to describe the Boolean functions: ∧ for conjunction, or logical AND; ∨ for disjunction, or logical OR and ¬ for negation, or logical NOT. Note that for *r*>1, the mRNA state space does not contain all the 2^*n*^ Boolean states. Denote the set of all the possibly allowed mRNA states for a specific pair of *n* and *r* by $M(n,r):=\{\chi_{i}\}$ (shorted by ). Then the following discussions are for the mRNA states in  only. 

1. **Entry**: A new ribosome may attach the leftmost of the mRNA if and only if the first *r* codons are free. For the mRNA states in , this condition is equivalent to that the *r*th codon is empty since such mRNA states with the *r*th codon being empty and any codon between 1 to *r*−1 being occupied are not allowed. This may be succinctly encoded in Boolean terms. The Boolean function for the entry event ${\vec{f}}_{0}$ can thus be written as follows,

(20) $$\begin{array}{l} {\vec{f}}_{0}:\left\{\begin{array}{ll} x_{1}(t+1) & =\neg x_{r}(t)\vee x_{1}(t)\\ & \vdots \\ x_{r}(t+1) & =\neg x_{r}(t)\vee x_{r}(t)\\ x_{r+1}(t+1) & =x_{r+1}(t)\\ & \vdots \\ x_{n}(t+1) & =x_{n}(t) \end{array}\right) \end{array}$$

The Boolean function ${\vec{f}}_{0}$ ensures that the first *r* codons will be occupied (a new ribosome enters) at time *t*+1 if the *r*th codon is empty (the first *r* codons are empty) at time *t*, and the first *r* codons keep unchanged (no ribosome enters) at time *t*+1 if the *r*th codon is occupied (a ribosome is present somewhere that prevents a new ribosome to enter) at time *t*. Therefore, this Boolean function agrees with the dynamics of the entry event *e*_0_ in Algorithm 6.

2. **Exit**: A ribosome dissociates from the rightmost of the mRNA if and only if the last *r* codons are occupied. For the mRNA states in , this condition is equivalent to that the last codon is occupied since such mRNA states with the last codon being occupied and any of the codons from *n*−*r*+1 to *n*−1 being empty are not allowed. The Boolean function for the exit event ${\vec{f}}_{n}$ can thus be written as follows,

(21) $$\begin{array}{l} {\vec{f}}_{n}:\left\{\begin{array}{ll} x_{1}(t+1) & =x_{1}(t)\\ & \vdots \\ x_{n-r}(t+1) & =x_{n-r}(t)\\ x_{n-r+1}(t+1) & =\neg x_{n}(t)\wedge x_{n-r+1}(t)\\ & \vdots \\ x_{n}(t+1) & =\neg x_{n}(t)\wedge x_{n}(t) \end{array}\right) \end{array}$$

The Boolean function ${\vec{f}}_{n}$ ensures that the last *r* codons will be empty (the ribosome dissociates from the mRNA) at time *t*+1 if the last codon is occupied (a ribosome is ready to exit) at time *t*, and the last *r* codons will keep unchanged at time *t*+1 if the last codon is empty (no ribosome is ready to exit) at time *t*. Therefore, this Boolean function agrees with the dynamics of the exit event *e*_*n*_ in Algorithm 6.

3. **Hops**: A ribosome with its head at codon *j* can move one codon towards its right if and only if the codons from *j*−*r*+1 to *j* are occupied and codon *j*+1 is empty. For the mRNA states in , this condition is equivalent to that codon *j* is occupied and codon *j*+1 is empty since such mRNA states with codon *j* being occupied, codon *j*+1 being empty and any of the codons between *j*−*r*+1 to *j*−1 being empty are not allowed. The Boolean function for the hopping event from codon *j* to *j*+1, ${\vec{f}}_{j}$, can then be written as follows,

(22) $$\begin{array}{l} {\vec{f}}_{j}:\left\{\begin{array}{ll} x_{1}(t+1) & =x_{1}(t)\\ & \vdots \\ x_{j-r}(t+1) & =x_{j-r}(t)\\ x_{j-r+1}(t+1) & =(\neg x_{j}(t)\vee x_{j+1}(t))\wedge x_{j-r+1}(t)\\ x_{j-r+2}(t+1) & =x_{j-r+2}(t)\\ & \vdots \\ x_{j}(t+1) & =x_{j}(t)\\ x_{j+1}(t+1) & =(x_{j}(t)\wedge \neg x_{j+1}(t))\vee x_{j+1}(t)\\ x_{j+2}(t+1) & =x_{j+2}(t)\\ & \vdots \\ x_{n}(t+1) & =x_{n}(t) \end{array}\right) \end{array}$$

The Boolean function ${\vec{f}}_{j}$ ensures that codon *j*−*r*+1 will be empty and codon *j*+1 will be occupied (a ribosome with its head at codon *j* hops one codon ahead) at time *t*+1 if codon *j* is occupied and codon *j*+1 is empty (a ribosome with its head at codon *j* can hop one codon ahead) at time *t*, and the codons from *j*−*r*+1 to *j*+1 will keep unchanged (no hopping event *e*_*j*_ occurs) at time *t*+1 if either codon *j* is empty or codon *j*+1 is occupied (no ribosme with its head at codon *j* is ready to hop one codon ahead) at time *t*. Therefore, this Boolean function agrees with the dynamics of the hopping event *e*_*j*_ in Algorithm 6.

The above Boolean functions thus constitute the Boolean descriptions of the update events, $F_{n,r}:=\{{\vec{f}}_{i},i\in I_{e}\}$. From the semi-tensor product theory, each Boolean function ${\vec{f}}_{i},i\in I_{e}$ is equivalent to a structure matrix $L_{i},i\in I_{e}$, thus making a set of structure matrices ${ℒ}_{n,r}:=\{L_{i},i\in I_{e}\}$ being a complete description of the update events. Notice that the Boolean functions determined here are independent of the time steps and the event occurrence probabilities.

#### The PBN model for mRNA translation

According to Algorithm 6, the next update event is selected probabilistically, and therefore the update events, i.e., the structure matrices ${ℒ}_{n,r}$ are associated with the corresponding probabilities $P_{n,r}:=\{p_{i}^{d_{2}}|i\in I_{e}\}$ as given in (12). Then the dynamics of the mRNA state can be described by a PBN, as follows,

$$x(t+1)=L_{x}x(t),x\in M$$

where $P\{L_{x}=L_{i}\}=p_{i}^{d_{2}},i\in I_{e}$.

From the PBN theory [82], the mean dynamics governed by the above PBN model is of the form $\langle x(t+1)\rangle =L_{E}\langle x(t)\rangle ,x(t)\in M$ and the stationary mean value of *x*, satisfies *y*=*L*_*E*_*y* where the probabilistic transition matrix is given by

(23) $$L_{E}:=\underset{i\in I_{e}}{\sum }p_{i}^{d_{2}}L_{i}$$

That is, *L*_*E*_ is the average over all the possible update events, weighted by their update probabilities.

Further, if we notice that for all x∉M, they are not governed by the dynamics in (23) and do not affect the system behaviour, then (23) can be reduced to the states in  only. The transition matrix *M*_*E*_ of the reduced system is obtained by deleting from *L*_*E*_ all the rows and columns that do not belong to ,

(24) $$\begin{array}{l} M_{E}=L_{E}{|}_{\text{Row}(L_{E})\in M,\text{Col}(L_{E})\in M} \end{array}$$

The reduced system is a Markov chain, where the state *i* in the Markov chain is *χ*_*i*_ in the original system. *M*_*E*_ can also be obtained from the reduced structure matrix for each event, $M_{i}=L_{i}{|}_{\text{Row}(L_{i})\in M,\text{Col}(L_{i})\in M}$ in a similar way as *L*_*E*_: $M_{E}:=\underset{i\in I_{e}}{\sum }p_{i}M_{i}$.

#### Modeling added biological complexities with the PBN model

The PBN model can be accommodated with added biological complexities, as long as the added complexity can be represented as Boolean functions. A new set of Boolean functions and transition probabilities can then be obtained and consequently the PBN model is constructed without any particular difficulty. An example is shown as follows. 

• Modelling premature stop codon. At the premature stop codon *j*, the ribosome can readthrough and then proceed to the production of the full-length protein, which is a normal hopping event described by the Boolean function ${\vec{f}}_{j}$. The ribosome can also dissociate from codon *j*, which is a new update event, whose Boolean function is denoted by ${\vec{f}}_{j_{d}}$. This Boolean function can be written as follows,

(25) $$\begin{array}{l} {\vec{f}}_{j^{d}}:\left\{\begin{array}{ll} x_{1}(t+1) & =x_{1}(t)\\ & \vdots \\ x_{j-r}(t+1) & =x_{j-r}(t)\\ x_{j-r+1}(t+1) & =(\neg x_{j}(t)\wedge x_{j+1}(t))\wedge x_{j-r+1}(t)\\ & \vdots \\ x_{j}(t+1) & =(\neg x_{j}(t)\wedge x_{j+1}(t))\wedge x_{j}(t)\\ x_{j+1}(t+1) & =x_{j+1}(t)\\ & \vdots \\ x_{n}(t+1) & =x_{n}(t) \end{array}\right) \end{array}$$

• The Boolean function ${\vec{f}}_{j_{d}}$ ensures that the codons from *j*−*r*+1 to *j* will be empty (a ribosome with its head at codon *j* dissociates from the mRNA) at time *t*+1 if codon *j* is occupied and codon *j*+1 is empty (the premature stop codon is occupied by the head of a ribosome) at time *t*, and the codons from *j*−*r*+1 to *j* will keep unchanged if either codon *j* is empty or codon *j*+1 is occupied (the premature stop codon is not occupied by the head of a ribosome) at time *t*. Therefore, this Boolean function agrees with the dynamics of the premature termination event $e_{j_{d}}$.

• Then, to model the premature stop codon with the PBN model, an extra Boolean function corresponding to the premature termination event is added to the set of the structure matrices, with also modified event probabilities for the premature termination event and the readthrough event. The PBN model can be constructed as usual with these modified set of structure matrices and corresponding probabilities. No particular difficulties are caused for the PBN model by the introduction the premature stop codon.

## Competing interests

The authors declare that they have no competing interests.

## Authors’ contributions

JK and YBZ planned the paper, YBZ performed the computations and YBZ and JK wrote the paper. Both authors read and approved the final manuscript.

## Supplementary Material

Additional file 1 Supplementary material. Click here for file
