# Supplementary material for: mRNA translation and protein synthesis: an analysis of different modelling methodologies and a new PBN based approach
Source: BMC Syst Biol. 2014 Feb 27;8:25. doi: 10.1186/1752-0509-8-25 (PMC4015640; doi:10.1186/1752-0509-8-25)
Supplement: Additional file 1 — Supplementary material. [file 1752-0509-8-25-S1.docx]

**Supplementary material**

In this section, we briefly discuss some additional details related to material presented in the text.

The first point relates to simulations performed with an increased number of codons. We performed simulations with 120 codons and the steady state was obtained from simulations with Algorithm 1 for the random-sequentially updated TASEP and analytically/numerically computed for the Heinrich model. The parameters are n=120, r=12, $\beta$=$\gamma$=0.5. The simulation procedure was the same as other simulations that used Algorithm 1.

Simulations with 120 codons in fact show very similar trends as the case with fewer codons. The translation rate and the codon densities increase with the increase of the initiation rate, but they become saturated fairly quickly, and this trend is exactly like what has been shown in Figure 2 for fewer codons.

**For all the plots**

- The parameters used were mentioned in the ‘caption’ field of each figure.  They can be found at the end of the paper.
- The methods used for each figure are as follows.

| Figure 2 | Heinrich model,  standard random-sequential TASEP, Petri net |
| --- | --- |
| Figure 3 | standard random-sequential TASEP, standard parallel TASEP |
| Figure 4 | standard random-sequential TASEP, standard parallel TASEP, standard PBN, Petri net |
| Figure 5 | random-sequential TASEP, parallel TASEP, PBN, all modified |
| Figure 6 | random-sequential TASEP with feedback |
|  |  |

- **Heinrich model.** Analysis of the Heinrich model relates initiation, elongation, termination and steady state translation rates. The formula used was equation (6) in Heinrich’s original paper: *J. Theor. Biol.,* **1980**, Vol.86(2), 279-313.
- **Petri net model.** All the calculations, for the initiation rates (Figure 2) or elongation rates (Figure 4) to the translation rates were based on TABLE 1 in the original paper: *J. Theor. Biol.,* **2012**, Vol.303, 128-140. This in turn is based on analytical results, which were checked against computational results. The essential conclusion from the analysis of this model was basically that the translation rate was determined by the slowest event rate (either initiation, elongation or termination) in a rather direct manner, which is somewhat different from the other models. In the paper, we have accounted for the multiple codon coverage as discussed in the text, and employed the essential results with the modification mentioned.
- **PBN.** All the calculations were direct analytical/numerical, without any simulations, based on Algorithm 3. With this algorithm, the stationary distribution is directly calculated from the given event rates.
- **Standard random-sequential TASEP,** all simulated based on Algorithm 1. **Standard parallel TASEP**, all simulated based on Algorithm 2. **Random-sequential TASEP with feedback.** It is modified from Algorithm 1.  The difference from the standard random-sequential TASEP is that the initiation rate is now time-varying, but as long as the initiation rate is known, then each update step is simulated exactly as in Algorithm 1. Within each update time interval, the change of the protein concentration is recorded. This change is due to two factors, the production of the protein (the number of protein increases by 1 if a termination event is triggered), and the degradation of the proteins. The change of the protein concentration then leads to the update of the initiation rate as in (8), and the next update event is simulated based on this new set of event rates. As mentioned in the text, the steady state can also be obtained from the PBN formulation with an extra equation which accounts for the feedback.
